# Carbon Nano-Onions as Non-Cytotoxic Carriers for Cellular Uptake of Glycopeptides and Proteins

**DOI:** 10.3390/nano9081069

**Published:** 2019-07-25

**Authors:** Marta d’Amora, Viviana Maffeis, Rosaria Brescia, Danielle Barnes, Eoin Scanlan, Silvia Giordani

**Affiliations:** 1 Nano Carbon Materials, Istituto Italiano di Tecnologia (IIT), Via Livorno, 60, 10144 Turin, Italy; 2 Electron Microscopy Facility, Istituto Italiano di Tecnologia (IIT), Via Morego 30, 16163 Genoa, Italy; 3 School of Chemistry and Trinity Biomedical Sciences Institute (TBSI), Trinity College Dublin, The University of Dublin, D02 P902 Dublin, Ireland; 4 School of Chemical Sciences, Dublin City University (DCU), Glasnevin, D09 C7F8 Dublin, Ireland

**Keywords:** carbon nano-onions, biomolecules, scaffolds, carriers, biomedical applications

## Abstract

Carbon nano-onions (CNOs) possess favorable properties that make them suitable for biomedical applications, including their small size, ready surface modification, and good biocompatibility. Here, we report the covalent immobilization of a synthetic glycopeptide and the protein bovine serum albumin (BSA) onto the surface of carbon nano-onions using the maleimide–thiol “addition reaction”. The glycopeptide and BSA are readily transported inside different cell lines, together with carbon nano-onions, through the endocytosis pathway. Our results show that carbon nano-onions are excellent scaffolds for glycopeptides and proteins immobilization and act as intracellular carriers for these biomolecules. These findings open new perspectives in the application of carbon nano-onions as intracellular transporters in diverse biomedical applications.

## 1. Introduction

Carbon nanomaterials (CNMs) are excellent materials for biomedical applications, including biological imaging and delivery of drugs, genes, and therapeutic agents [1,2,3]. CNMs have a small, tunable size distribution (1–100 nm), comparable to that of key biomolecules (DNA and proteins). The size of the CNMs is an important feature in their ability to function as carriers for cellular uptake of therapeutic payloads. As a result of their small size and ability to be easily functionalized, CNMs are excellent intracellular transporters [4,5]. Within this context, carbon nano-onions (CNOs) offer several beneficial properties that make them a suitable material for biomedical and related applications. CNOs are sufficiently small to be carried easily through the circulatory system, and their high surface area allows surface modification and multi-functionalization. They have a relatively higher intracellular uptake and broader availability to a range of biological targets owing to their small size and increased mobility [6,7,8]. Moreover, our group has reported a high in vivo biocompatibility of oxidized carbon nano-onions, carbon nano-onions functionalized with a green fluorophore [9,10], and CNOs low inflammatory potential [11]. We have shown the ability of carbon nano-onions functionalized with different fluorophores to enter into several healthy and cancer cell lines with a high cellular uptake efficiency [12,13]. We have demonstrated that fluorescent carbon nano-onions are suitable nanoprobes for diagnostics [14] and platforms for imaging [15]. Different covalent functionalization strategies have been employed so far to decorate the surface of CNOs [6]. In particular, amidation and esterification reactions of oxidized CNOs have been reported for the successful attachment of bioactive moieties [8]. They can also be conjugated with biomolecules, such as peptides, glycopeptides, and proteins, opening new perspectives for their applications as intracellular carriers for biomolecules. Recently, significant focus has been placed on the development of mild ligation strategies suitable for the precise construction of protein-nanomaterial conjugates [2,16]. An efficient strategy for bioconjugation involves the chemical introduction of an electrophilic maleimido group onto the surface of the nanomaterial via a covalently bound linker. The maleimido group can undergo chemoselective reactions with the thiol moiety of a cysteine residue displayed on the surface of a peptide or protein in order to furnish the thioether linked protein/peptide. Here we report the first examples of covalent modification of CNOs decorated with maleimido groups suitable for chemoselective ligation with thiol-containing biomolecules. In particular, fluorescein-labeled glycopeptide and bovine serum albumin (BSA) were respectively immobilized onto the surface of CNOs. Following successful covalent ligation, we investigated the novel nanoconjugates in terms of toxicological profile and uptake efficiency. An important finding of our work is that a fully protected glycopeptide, that has significantly different polarity to an unprotected system and that offers the potential to function as a pro-drug, was successfully uptaken by cells when carried by the CNO. Glycopeptide-CNOs conjugates (Gly-CNOs) and bovine serum albumin-CNOs conjugates (BSA-CNOs) were readily uptaken by both healthy and cancer cells in a short time by endocytosis pathways. The internalized Gly-CNOs and BSA-CNOs presented good biocompatibility and localized partially in the lysosomes. In contrast, the synthetic glycopeptide alone was not able to enter in the cells, and the pure BSA was internalized with a lower efficacy compared to the substrate covalently bound to the carbon nano-onion carriers. Our results clearly demonstrated that carbon nano-onions enabled or enhanced the internalization of the fluorescent synthetic glycopeptide and BSA in cells, suggesting their potential as glycopeptides and protein carriers.

## 2. Materials and Methods

### 2.1. Synthesis and Functionalization of Carbon Nano-Onions (CNOs)

#### 2.1.1. p-CNOs

The synthesis of small CNOs (6–8 shells) was performed by annealing nanodiamond powder (Molto, 5 nm average particle size) under a positive pressure of helium at 1650 °C [17,18].

#### 2.1.2. ox-CNOs

Through treatment of these pristine CNOs (100 mg) in 75 mL of a solution of nitric acid (3 M) stirring under reflux for 48 h, defective sites on the surface were transformed into carboxylic acid by oxidation. After cooling to room temperature (RT), the CNOs were filtered off on a nylon filter membrane (pore size 0.2 μm) and washed with water, methanol, and acetone. ox-CNO (99 mg) was recovered as black powder after drying overnight at RT.

#### 2.1.3. PEG-CNOs

The carboxylic groups of ox-CNOs (80 mg) were amidated with 4,7,10-trioxa-1,13-tridecanediamine (15 mL) in dry *N,N*-dimethylformamide (DMF) (15 mL) by water-soluble 1-(3-thylaminopropyl)-3-ethylcarbodiimide hydrochloride (EDC HCl) and 4-(dimethylamino) pyridine (DMAP) while stirring the ox-CNOs under reflux at 140 °C for 4 d. After complexation, the CNOs were filtered off on a nylon filter membrane (pore size 0.2 μm) and washed with water, methanol, and acetone. PEG-CNO (163 mg) was recovered as black powder after drying overnight at RT.

#### 2.1.4. Maleimido Decorated CNOs (Mal-CNOs)

PEG-CNOs (20 mg) was solubilized in dry DMF (10 mL), and 120 mg of maleic anhydride was added. The solution was sonicated for 20 min, heated at 75 °C for 2 h, and stirred at room temperature for 16 h. The reaction was filtered off on a nylon filter membrane (pore size 0.2 μm) and washed with water and tetrahydrofuran (THF) to remove unreacted compounds.

#### 2.1.5. Glycopeptide-CNO Conjugates (Gly-CNOs)

PEG-mal-CNO (10 mg) was dispersed in dry DMF (4 mL), and 20 mg of fluorescently labeled glycopeptide 1 was added under nitrogen. The solution was sonicated 20 min and the pH adjusted to 7.4 with NaHCO_3_. The reaction was stirred for 16 h at room temperature in the dark. After complexation, the solution was filtered off on a nylon filter membrane (pore size 0.2 μm) and washed with MeOH and acetone. The material obtained was re-dispersed in MeOH, filtered again, and centrifuged 3 times in MeOH at 5000 rpm for 10 min to wash out the unbound species.

#### 2.1.6. Bovine Serum Albumin-CNO Conjugates (BSA-CNOs)

PEG-mal-CNO (6 mg) was solubilized in dry DMF (5 mL), and 12 mg of BSA-FITC was added in H_2_O (5 mL) under nitrogen. The solution was sonicated 20 min and the pH adjusted to 7.4 with NaHCO_3_. The reaction was stirred for 16 h at room temperature in the dark. After ligation, the solution was filtered on nylon and washed with MeOH and acetone. The material obtained was re-dispersed in MeOH, filtered again, and centrifuged 3 times in MeOH at 5000 rpm for 10 min to wash out the unbound species. The BSA-CNO conjugate was further characterized by TGA, FTIR, absorption and emission spectroscopy, Z-potential, and dynamic light scattering (DLS) measurements.

#### 2.1.7. Transmission Electron Microscopy (TEM) Analysis of CNOs

High-resolution TEM (HR-TEM) analyses were carried out by an image-Cs-corrected (CEOS GmbH, Heidelberg, Germany) JEM-2200FS microscope (JEOL, Tokyo, Japan),), operated at 200 kV and equipped with a Bruker XFlash 5060 SDD system for energy-dispersive spectroscopy (EDS, Bruker Nano GmbH in Berlin, Germany). For these analyses, the CNOs were suspended in ethanol and sonicated, then a few microliters of each suspension was drop-cast onto a lacey carbon-film-coated Cu grid.

### 2.2. Biological Studies

#### 2.2.1. Cell Culture

Mouse embryonic fibroblast cells (NIH 3T3) and human breast adenocarcinoma cells (MCF7) were maintained in Dulbecco’s modified Eagle’s medium (Life Technologies), supplemented with 10% fetal bovine serum (Life Technologies, Inc Grand Island, NY), and 1% penicillin and streptomycin (Sigma Aldrich, St. Louis, MO, USA). Cells were grown at 37 °C with 5% CO_2_ and 95% air.

#### 2.2.2. Cytotoxicity Analysis

NIH 3T3 and MCF7 cells were seeded at a concentration of 5000 cells/well and maintained in an incubator overnight at 37 °C with 5% CO_2_. ox-CNOs, Gly-CNOs, BSA-CNOs, glycopeptide 1, and BSA-FITC were suspended in medium at final concentrations of 10 and 20 μg mL^−1^, followed by sonication for 15 min at 37 kHz. The maintenance medium was removed and replaced with 500 μL of ox-CNOs, Gly-CNOs, BSA-CNOs, glycopeptide 1, or BSA-FITC solutions. As a positive control, cells were treated with 5% DMSO. Cell viability was determined after 24, 48, and 72 h of exposure to CNO-bioconjugates, glycopeptide 1, and BSA-FITC, using the WST1 assay (Roche Applied Science, Indianapolis, IN, USA). Data were expressed as the mean ± standard deviation (mean ± SD).

#### 2.2.3. Cellular Uptake Mechanism

For uptake studies, NIH 3T3 cells and MCF7 cells were seeded in nunc chambers and incubated overnight. After this time, the medium was removed, and cells were treated with various concentrations of BSA-CNOs or BSA-FITC for 24 h and of Gly-CNOs and glycopeptide 1 for 2, 6, 12, 16, and 24 h. As a negative control, cells were maintained in cell medium. After the different incubations, the cells were washed three times with PBS and then treated with a solution of Hoechst 33342 (Sigma Aldrich, St. Louis, MO, USA) or Hoechst 33342 (Sigma Aldrich, St. Louis, MO, USA) with Lysotracker Deep Red dye (Life Technologies, Inc Grand Island, NY, USA) for 15 min. After incubation, the cells were washed three times with PBS and observed with a confocal microscope (Nikon A1, Tokyo, Japan).

## 3. Results

### 3.1. Synthesis of Gly-CNOs and BSA-CNOs

**Scheme 1:**
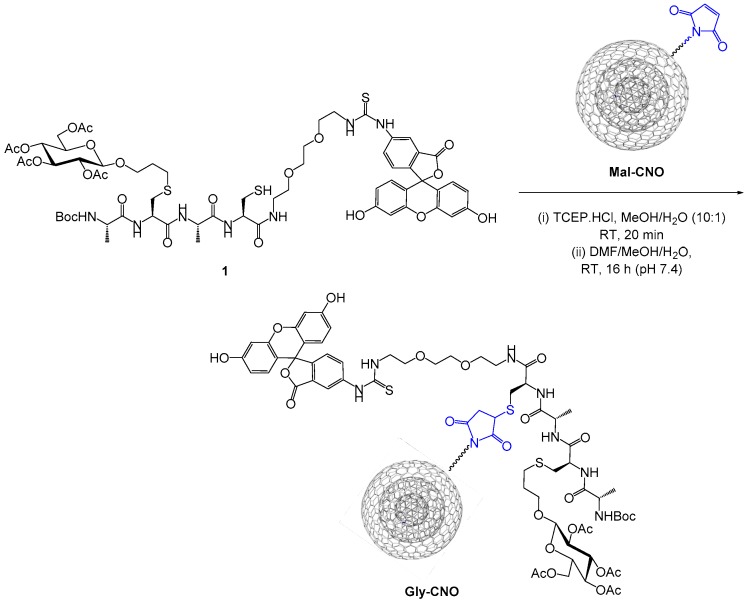
Ligation reaction between synthetic glycopeptide 1 and maleimido decorated CNO (Mal-CNO).

Maleimido decorated CNOs (Mal-CNO) suitable for covalent modification with cysteine-containing substrates were prepared through a multistep protocol starting from nanodiamonds (see Supplementary Information). With the Mal-CNO in hand, we set out to investigate the ligation of a synthetic, cysteine-containing glycopeptide onto the CNOs. The structure of glycopeptide 1 was based on our previously reported ala-cys-ala-cys tetrapeptide core [19]. The glycopeptide was prepared through a sequential native chemical ligation, thiol-ene addition (NCL-TEC) approach. A protected galactose reside was S-linked onto the peptide backbone using a thiol-ene ligation reaction between a cysteine residue and a modified glycan, displaying a terminal alkene residue at the anomeric position. A second cysteine residue was subsequently introduced through native chemical ligation to provide a reactive moiety for subsequent maleimido coupling. The glycopeptide was modified at the C-terminus with a short, fluorescein-labeled PEG-group to furnish the fluorescently labeled glycopeptide 1 (see Supplementary Information for detailed synthetic procedures and compound characterization). The coupling reaction was carried out using *N,N*′-diisopropylcarbodiimide (DIC)/(1-hydroxybenzotriazole (HOBt) mediated conditions, which are compatible with the sulfhydryl group. For the ligation reaction, Mal-CNO was treated at pH 7.4 with glycopeptide **1**. The reaction was stirred for 16 h at room temperature in the dark. The modified CNOs were filtered off on a nylon filter membrane (pore size 0.2 μm) and washed with MeOH and acetone. The resulting material was re-dispersed in MeOH, filtered again, and centrifuged in MeOH to wash out any noncovalently bound species (Scheme 1).

**Scheme 2:**
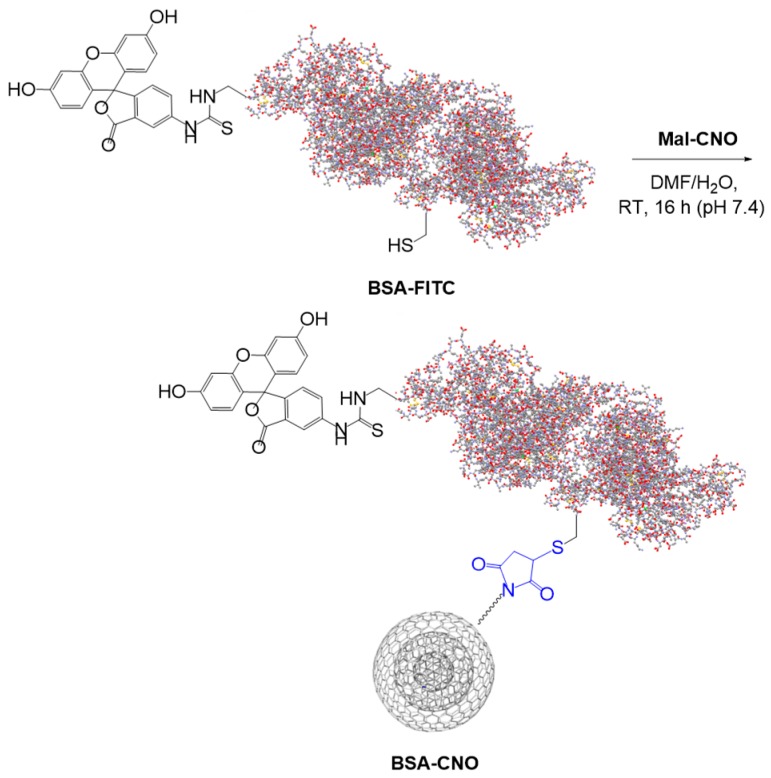
Ligation reaction between BSA-FITC and Mal-CNO.

Covalent modification of nanomaterials with proteins represents an effective method for modifying biocompatibility. In order to investigate the maleimide-mediated ligation methodology for covalent protein modification, we carried out the ligation reaction of Mal-CNO with BSA-FITC. Mal-CNO was treated with BSA-FITC for 16 h at pH 7.4 in buffer. On completion of the ligation reaction, the solution was filtered on nylon and washed with MeOH and acetone. BSA-CNO was redispersed in MeOH, filtered again, and centrifuged to wash out the unbound species (Scheme 2).

### 3.2. Characterization of Gly-CNOs and BSA-CNOs

**Figure 1:**
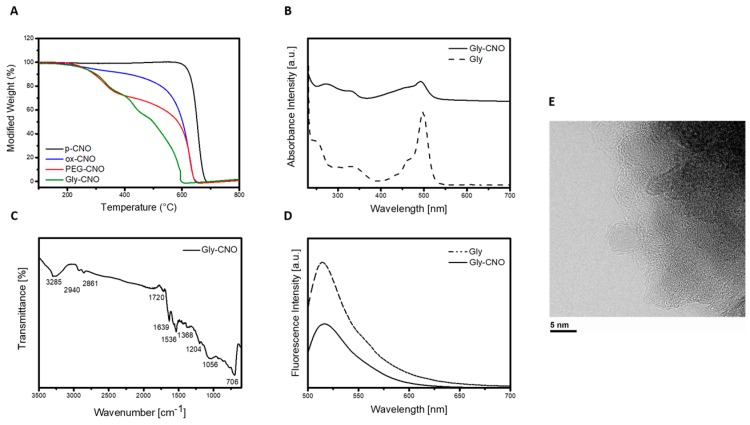
Characterization of the functionalized glycopeptide-CNO conjugate (Gly-CNO). (**A**) Thermogravimetric analysis of *p*-CNO (black), ox-CNO (blue), PEG-CNO (red), and Gly-CNO (green). All experiments were run in air with a temperature rate of 10 °C min^−1^. (**B**) Absorption spectra of Gly-CNO (solid line) and fluorescent labeled glycopeptide 1 (dotted line) in DMEM phenol red free at a concentration of 10 μg mL^−1^. (**C**) ATR-FTIR spectra of Gly-CNO using germanium crystals and 256 scans. (**D**) Emission spectra (λ_exc_ = 490 nm) of fluorescent labeled glycopeptide 1 (dotted line) and Gly-CNO (solid line) in DMEM at a concentration of 10 μg mL^−1^. (**E**) HR-TEM image of a portion of a cluster of Gly-CNOs, partly suspended on a hole in the carbon film.

Evidence for the successful covalent modification of Mal-CNO with glycopeptide **1** was provided by nuclear magnetic resonance (NMR), ATR-FTIR, TGA, UV-spectroscopy, and emission analysis. In the NMR spectrum of the conjugate, peaks corresponding to the methyl groups of the alanine residues, Boc protecting group, acetyl group, PEG, and FITC moieties were clearly visible (see Supplementary Information). FTIR analysis of the Gly-CNO sample showed the characteristic vibration bands of broad O–H centered at (3285 cm^−1^), N–H (2940 cm^−1^), S–H (2861 cm^−1^), C=O and amides (1720, 1639, and 1536 cm^−1^), C=S (1368 cm^−1^), and COC (1056 cm^−1^) (Figure 1c). TGA performed in air showed thermal decomposition of surface organics at 330 °C and at 430 °C respectively, followed by decomposition of the CNO core at 510 °C (Figure 1a). The emission properties of the Gly-CNOs were investigated under physiological conditions. Upon photoexcitation at 490 nm in biological medium (Dulbecco’s modified Eagle’s medium, DMEM) without phenol red, an intense emission band was observed with a maximum at 518 nm (Figure 1d). The result was comparable to the emission of the free glycopeptide (λ_em_ = 516 nm), suggesting a similar microenvironment surrounded the fluorophores once the biomolecules were anchored onto the surface of the CNOs. The concentration of glycopeptide in a 10 μg mL^−1^ dispersion of Gly-CNO was determined to be about 4.6 × 10^−6^ M by absorption (Figure 1b).

The kinetic of aggregation and size of the clusters formed were investigated by dynamic light scattering (DLS). DLS measurements were performed on the CNO samples dispersed in water (see Supplementary Information). The hydrodynamic diameter was found to be 236 ± 4 nm and 325 ± 6 nm, respectively, for PEG-CNOs and Gly-CNOs at a concentration of 5 µg mL^−1^. The results were in agreement with a functionalized surface. HR-TEM analyses of clusters of Gly-CNOs showed an approximately spherical shape of the individual particles with typical concentric graphitic shells with 3.4 Å spacing (Figure 1e). The average diameter of an individual CNO was 6 ± 1 nm, and their successful labeling was confirmed by incorporation of S, N, and O, as detected by STEM-EDS analyses (see Supplementary Information) in this sample, to be compared to pristine CNOs. Furthermore, Z-potentials of p-CNO and Gly-CNO samples were measured in phosphate buffer 0.01 M at pH 7.4. Under these conditions, the Z-potential for the p-CNO was found to be −28.1 ± 1 mV, independently of the concentration used. Interestingly, after glycopeptide ligation, the Z-potential was found to be −36 ± 1 mV for Gly-CNOs, indicating that the biomolecules had a significant effect on the colloidal stability of the nanoparticles. Indeed, nanoparticles with Z-potential values above 25 mV (either positive or negative) have high degrees of stability [20]. The repulsion forces originating from the Gly-CNO surface charges prevent the particles from aggregating, whereas nanoparticles with a low zeta potential value could aggregate due to van der Waals interparticle attractions.

**Figure 2:**
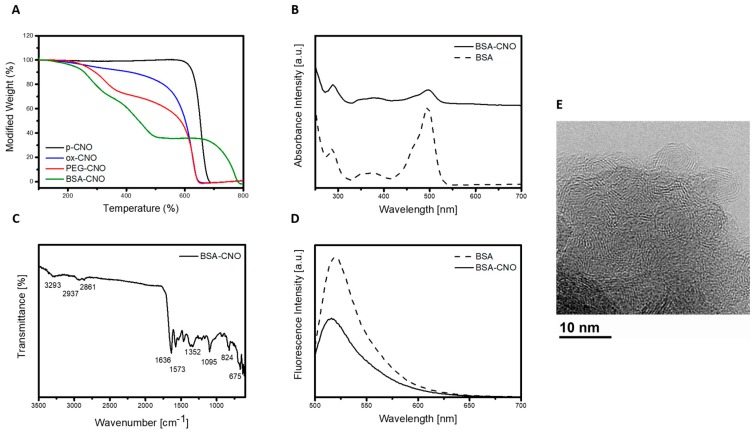
Characterization of the functionalized bovine serum albumin (BSA)-CNO. (**A**) Thermogravimetric analysis of p-CNO (black), ox-CNO (blue), PEG-CNO (red), and BSA-CNO (green). All experiments were run in air with a temperature rate of 10 °C min^−1^. (**B**) Absorption spectra of BSA-CNO (solid line) and BSA-FITC (dotted line) in DMEM phenol red free at a concentration of 10 μg mL^−1^. (**C**) ATR-FTIR spectra of BSA-CNO using germanium crystals and 256 scans. (**D**) Emission spectra (λ_exc_ = 490 nm) of BSA-FITC (dotted line) and BSA-CNO (solid line) in DMEM at a concentration of 10 μg mL^−1^. (**E**) HR-TEM image of a portion of a cluster of BSA-CNOs, partly suspended on a hole in the carbon film.

Evidence for the successful covalent modification of Mal-CNO with BSA-FITC was also provided by ATR-FTIR, TGA, UV-spectroscopy, and emission analysis. Strong stretching vibrations were recorded for BSA-CNO. The main peaks showed the stretching vibration of OH at 3293 cm^−1^, amide A (mainly –NH stretching vibrations) at 2937 cm^−1^, amide I (mainly C=O stretching vibrations) at 1636 cm^−1^, and amide II (the coupling of bending vibrate of N–H and stretching vibrate of C–N) bands at 1573 cm^−1^. TGA performed in air displayed the thermal decomposition of surface-bound organics at 280 °C and at 440 °C, followed by decomposition of the CNO core at 720 °C (Figure 2a). Furthermore, upon photoexcitation at 490 nm in biological medium (DMEM) without phenol red, an intense emission band was observed with a maximum at 518 nm (Figure 2d). The result was comparable to the emission of the free BSA-FITC (λ_em_ = 518 nm), suggesting a similar microenvironment surrounded the fluorophores once the biomolecules were anchored onto the surface of the CNOs. The results, including the HR-TEM analyses (Figure 2e), were in close agreement with Gly-CNO discussed above, in terms of diameter of individual CNOs (6 ± 1 nm) and incorporation of S, N, and O (see EDS spectra in Supplementary Information). The concentration of the BSA in a 10 μg mL^−1^ dispersion of BSA-CNO was determined to be about 1.7 × 10^−4^ M. The hydrodynamic diameter was found to be 236 ± 4 nm and 705 ± 70 nm for PEG-CNOs and BSA-CNOs, respectively, at a concentration of 5 µg mL^−1^ (see Supplementary Information). Because DLS measures the scattered intensity over a range of scattering angles for a given time, it is notable that when the density of the protein is high, the strong influence of bioconjugate interactions becomes important. Despite that, after bioconjugation, the Z-potential was found to be −35 ± 2 mV. These observations were consistent with the results demonstrated for Gly-CNO.

### 3.3. Cytotoxicity Analysis of Nanoconjugates

**Figure 3:**
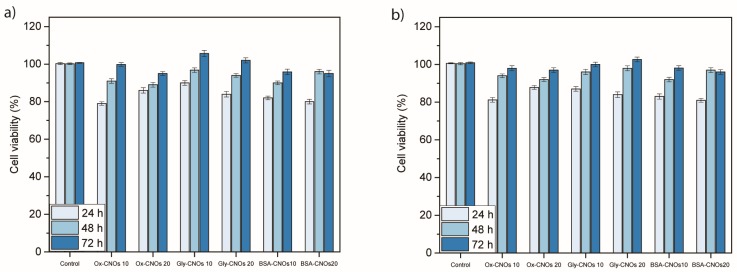
Cellular viability of (**a**) NIH 3T3 and (**b**) MCF7 cells treated with different concentrations (10 and 20 µg mL^−1^) of ox-CNOs, Gly-CNOs, and BSA-CNOs for 24, 48, and 72 h, revealed by WST 1 assay. Viability (%) is evaluated for the samples of CNOs against a nontreated control (ctrl). Data are expressed as mean ± standard error as calculated from three separate experiments.

In addition to structural studies on CNOs modified with BSA-FITC and glycopeptide **1**, the potential application of Gly-CNOs and BSA-CNOs in nanomedicine was evaluated by assessing their in vitro toxicities. The toxicological profiles of ox-CNOs, Gly-CNOs and BSA-CNOs were evaluated using one set of normal cells (NIH 3T3) and one set of cancer cells (MCF-7). Figure 3 shows the results of the WST1 assay after 24, 48, and 72 h of incubation with ox-CNOs, Gly-CNOs and BSA-CNOs. Once internalized on both cell lines, ox-CNOs and protein/glycopeptide-coated CNOs did not have adverse effects, showing high cell viabilities.

**Figure 4:**
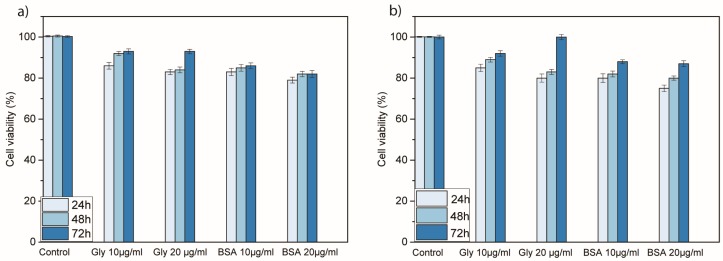
Cellular viability of (**a**) NIH 3T3 and (**b**) MCF7 cells treated with different concentrations (10 and 20 µg mL^−1^) of glycopeptide 1 and BSA-FITC for 24, 48, and 72 h, revealed by WST 1 assay. Viability (%) is evaluated for the samples of peptides against a nontreated control (ctrl). Data are expressed as mean ± standard error as calculated from three separate experiments.

For comparison, we also performed a toxicity assessment of the pure glycopeptide 1 and BSA-FITC. Cell viability of NIH 3T3 cells (Figure 4a) and MFC7 cells (Figure 4b) exposed to different concentrations of glycopeptide 1 and BSA-FITC were determined in the same conditions of the nanoconjugates. The results suggested that cell viability was unaffected by the internalization of pure glycopeptide and protein even after prolonged exposure.

### 3.4. Cellular Uptake of Nanoconjugates

#### 3.4.1. Cellular Internalization and Localization of Gly-CNOs

**Figure 5:**
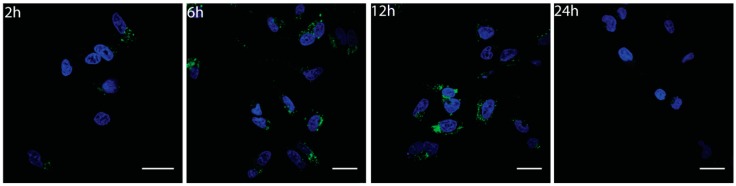
Fluorescent images of 3T3 cells incubated with Gly-CNOs at a mass concentration of 20 µg mL^−1^ for 2, 6, 12, and 24 h. Nuclei are stained with Hoechst 33342 (blue). Scale bars = 20 µm.

To investigate cellular uptake and localization of Gly-CNOs, we treated the normal (NIH 3T3) and cancer (MCF7) cell lines with different concentrations of the conjugates (1, 5, 10, and 20 µg mL^−1^) and for different incubation times (2, 6, 12, and 24 h). As shown in Figure 5 and Supplementary Information, the Gly-CNOs were successfully and easily uptaken by the cells in a short time (after 2 h). Moreover, Gly-CNOs were mainly localized in the perinuclear region, and they accumulated progressively in the cytoplasm (6, 12 h). However, at 24 h of incubation, the fluorescent signal of Gly-CNOs disappeared completely. Gly-CNOs showed a similar behavior in both cell lines (data not shown).

To understand the disappearance of the FITC signal after 24 h of incubation, we investigated the fate of Gly-CNOs after internalization in both normal and cancer cells. Since endocytosis, compared to passive diffusion, is hindered at low temperatures, we incubated the cells treated with different concentrations of Gly-CNOs at 4 °C. In all of the tested conditions, Gly-CNOs were partially internalized in the cells, indicating that endocytosis was the uptake pathway of these conjugates (data not shown).

**Figure 6:**
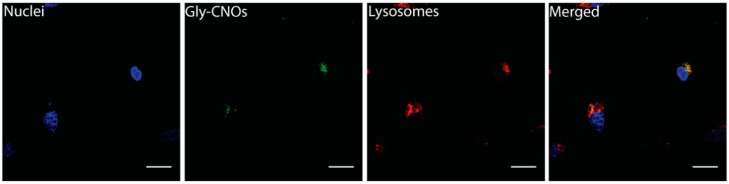
Fluorescent images of 3T3 cells incubated with Gly-CNOs at a mass concentration of 10 µg mL^−1^ for 16 h. Scale bars = 20 µm.

Moreover, we studied the cellular localization of Gly-CNOs by using LysoTracker Deep Red dye. We monitored intracellular localization from 2 h of exposure until 24 h with several time points (2, 6, 12, 16, and 24 h). Gly-CNOs started to localize in the lysosomes only around 12 h of incubation (Figure 6 and see also Supplementary Information), with a subsequent total co-localization with these organelles at 16 h. Our results indicated that Gly-CNOs ended up in the lysosomes. We postulate that following internalization in the lysosomes, Gly-CNOs were metabolized by the lysosomal enzymes, and this could explain the disappearance of the FITC signal at 24 h of incubation.

**Figure 7:**
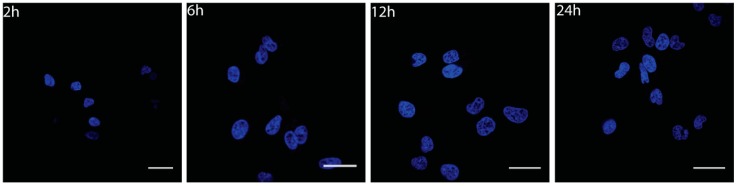
Fluorescent images of 3T3 cells incubated with glycopeptide 1 at a mass concentration of 5 µg mL^−1^ for 2, 6, 12, and 24h. Nuclei are stained with Hoechst 33342 (blue). Scale bars = 20 µm.

As control experiments, we treated the two cell lines with different concentrations of the fluorescent glycopeptide alone (1, 5, 10, and 20 µg mL^−1^) for the same temporal window (2, 6, 12, and 24 h). As shown in Figure 7 and in Supplementary Information, no fluorescent signal was detectable in all the tested concentrations and for all the temporal windows, indicating that the glycopeptide alone was unable to enter in the cells, and CNOs were successful in carrying the glycopeptide inside cells.

#### 3.4.2. Cellular Internalization and Localization of BSA-CNOs

**Figure 8:**
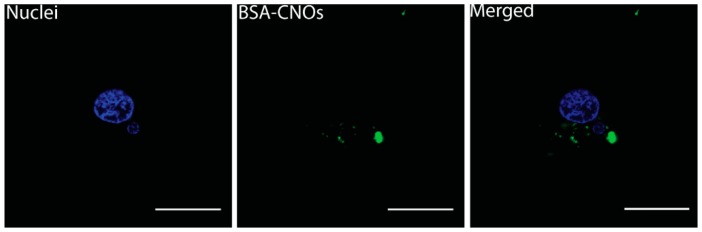
Fluorescent images of 3T3 cells incubated with BSA-CNOs at a mass concentration of 1 µg mL^−1^ for 24 h. Nuclei are stained with Hoechst 33342 (blue). Scale bars = 25 µm.

To confirm the ability of CNOs to function as carriers of biomolecules, we performed a second set of experiments using the BSA-CNOs. We incubated the cells with different concentration of BSA-CNO (1, 5, 10, and 20 µg mL^−1^) for 24 h. The nanoconjugates were internalized in the cell lines, even at a low concentration (Figure 8), in the perinuclear region with a point-like distribution.

As for the Gly-CNOs, we assessed the cellular localization of BSA-CNOs by decreasing the temperature during the incubation and by using LysoTracker Deep Red dye. At low temperatures (4 °C), BSA-CNOs were uptaken by the cells (data not shown), indicating that endocytosis was also the mechanism of uptake for these nanoconjugates.

**Figure 9:**
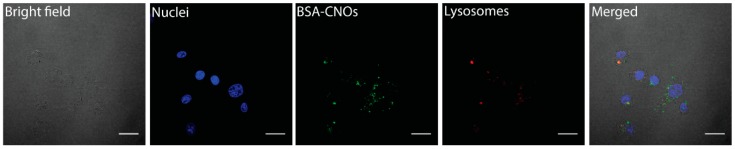
Fluorescent images of MCF-7 cells incubated with BSA-CNOs at a mass concentration of 1 µg mL^−1^ for 24 h. Scale bars = 20 µm.

We investigated the localization of BSA-CNOs with LysoTracker Deep Red dye. As shown in Figure 9, the nanoconjugates partially localized in the lysosomes.

**Figure 10:**
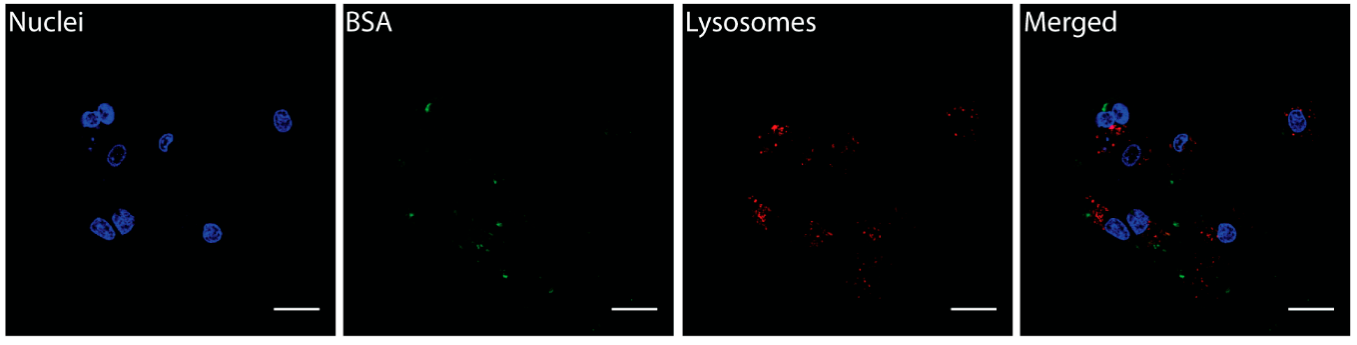
Fluorescent images of MCF-7 cells incubated with BSA-FITC at a mass concentration of 1 µg mL^−1^ for 24 h. Scale bars A–D = 20 µm.

As a control experiment, we treated the cells with different concentrations of pure BSA-FITC in the same experimental conditions. BSA-FITC alone was able to enter in cells but with lower efficacy with respect to when it was conjugated onto the carbon nano-onions (Figure 10). These findings clearly indicated that CNOs enhanced the uptake of BSA-FITC. Moreover, pure BSA did not localize in the lysosomes (no co-localization with LysoTracker Deep Red dye, Figure 10), while BSA-CNOs partially deposited on these organelles, suggesting a different mechanism of uptake of BSA-FITC alone and CNO conjugated with this protein.

## 4. Discussion

In summary, we developed a versatile ligation approach for the covalent modification of CNOs employing a chemoselective cysteine addition onto CNO-bound maleimide residues. We demonstrated for the first time the capability of carbon nano-onions to act as intracellular carriers of biomolecules, in particular for a synthetic glycopeptide and a model pure protein. Conjugation with CNOs enabled the synthetic glycopeptide to enter inside the cells. After 16 h, the nanoconjugates translocated and localized into lysosomes where they were metabolized, resulting in a complete quenching of fluorescence. Furthermore, conjugation of CNOs with BSA increased the uptake of the protein and altered the distribution of the protein within the cell, in comparison to the protein alone. The protein alone accumulated in the cytoplasm, while the protein conjugated with the CNOs translocated inside the lysosomes. Our results demonstrated that CNOs could act in both cases as carriers inside the cells, inducing the translocation of the transported biomolecules in the lysosomes. This mechanism did not lead to cell apoptosis. In fact, as shown by the cell viability, after translocation of the peptide and the protein, there were no adverse effects. However, altering the payload to a cytotoxic therapeutic could potentially induce this effect. Our results demonstrated that carbon nano-onions functioned as efficient carrier substrates to transport the biomolecules into the cells. After a temporal window of 16–24 h, CNOs translocated to the lysosomes to be subsequently metabolized without inducing any effects on the cells. These findings indicated that CNOs are able to carry a molecule inside a cell and expose it to lysosomal degradation after a precise temporal window during which the molecule can perform its specific function.

Our results open new perspectives in the application of carbon nano-onions in biomedicine. This finding offers fascinating prospects for the development of biocompatible nanomaterials, decorated with highly defined synthetic peptides and glycopeptides as novel therapeutics. Furthermore, this methodology can envisage the realization of multistimuli-responsive and dynamic materials capable of altering their physicochemical behavior upon encountering specific microenvironmental signals, which is becoming relevant for diagnosis and medical imaging applications.

## Supplementary Materials

The following are available online at https://www.mdpi.com/2079-4991/9/8/1069/s1, Scheme S1: Synthesis of functionalized fluorescein 2, Scheme S2: Synthesis of fluorescein-functionalized glycopeptide 1, Scheme S3: Synthesis of Maleimido-PEG conjugate 4, Scheme S4: Synthetic procedure for preparation of maleimido decorated CNOs, Figure S1: Absorption and Emission spectra (λexc = 495 nm) (bottom) of fluorescein-functionalized glycopeptide **1** at a concentration of 10 µg ml^−1^ in DMSO (top). FTIR spectroscopy of synthetic glycopeptide **1**, Figure S2: Absorption studies of fluorescent labelled glycopeptide **1** at a concentration of 5, 10, 15, 20 and 50 μg mL^−1^ in DMSO (top) and 1, 2, 4, 5, 6, 8 and 10 μg mL^−1^ in DMSO (bottom), Figure S3: Absorption studies of fluorescent labelled glycopeptide **1** at a concentration of 1, 2, 5, 6 and 10 μg mL^−1^ in DMEM phenol red free, Figure S4: Emission studies of fluorescent labelled glycopeptide **1** at a concentration of 5, 10, 15, 20 and 50 μg mL^−1^ in DMSO (top) and 1, 2, 4, 5, 6, 8 and 10 μg mL^−1^ in DMSO (bottom), Figure S5: Emission studies of fluorescent labelled glycopeptide **1** at a concentration of 1, 5, 6, 8 and 10 μg mL^−1^ in DMEM phenol red free, Figure S6: Absorption (top) and Emission spectra (λexc = 495 nm)(bottom) of **Gly-CNO** at 5, 10, 15, 20 and 50 μg mL^−1^ in DMEM-phenol red free, Figure S7: Absorption (top) and Emission spectra (λexc = 495 nm)(bottom) of **BSA-CNO** at 5, 10, 15, 20 and 50 μg mL^−1^ in DMEM-phenol red free, Figure S8: DLS of **PEG-CNO** (red) and **Gly-CNO** (green) at a concentration of 5 µg ml^−1^ in water (left) and Z-potential measurement of **PEG-CNO** (red) and **Gly-CNO** (green) at a concentration of 10 µg ml^−1^ in phosphate buffer 0.01 M at pH 7.4 (right), Figure S9: DLS of **PEG-CNO** (red) and **BSA-CNO** (green) at a concentration of 5 µg ml^−1^ in water (left) and Z-potential measurement of **PEG-CNO** (red) and **BSA-CNO** (green) at a concentration of 10 µg ml^−1^ in phosphate buffer 0.01 M at pH 7.4 (right), Figure S10: ^1^H and ^13^C NMR of Fluorescein-Functionalized Glycopeptide **1**, Figure S11: ^1^H and ^13^C NMR of Functionalized Fluorescein **2**, Figure S12: ^1^H and ^13^C NMR of compound **4**, Figure S13: ^1^H NMR of **Mal-CNO**,Figure S14: Fluorescent images of 3T3 cells incubated with **Gly-CNOs** at a mass concentration of 5 µg mL^−1^ for 2, 6, 12 and 24h, Figure S15: Fluorescent images of 3T3 cells incubated with **Gly-CNOs** at a mass concentration of 10 µg mL^−1^ for 2 h, Figure S16: Fluorescent images of 3T3 cells incubated with glycopeptide **1** at a mass concentration of 20 µg mL^−^^1^ for 2, 6, 12 and 24h.
