# Supplementary material for: Carbon Nano-Onions as Non-Cytotoxic Carriers for Cellular Uptake of Glycopeptides and Proteins
Source: Nanomaterials (Basel). 2019 Jul 25;9(8):1069. doi: 10.3390/nano9081069 (PMC6722779; doi:10.3390/nano9081069)
Supplement: Supplementary file 1 [file nanomaterials-09-01069-s001.pdf]

nanomaterials

Supplementary Materials: Carbon nano-onions as non-cytotoxic carriers for cellular uptake of glycopeptides and proteins

Marta d'Amora, Viviana Maffeis, Rosaria Brescia, Danielle Barnes, Eoin Scanlan and Silvia Giordani

Section S1. General methods and Instrumentation

For NMR spectra, a 400 MHz spectrometer was employed for ^{1}H (400.13 MHz) and ^{13}C (100.61 MHz) spectra, a 600 MHz spectrometer was employed for ^{1}H (600.13 MHz) and ^{13}C (150.90 MHz) spectra. Resonances δ, are in ppm units downfield from an internal reference in $CDCl_3$ (δH = 7.26 ppm, δC = 77.0 ppm), or MeOH (δH = 3.31 ppm, δC = 49.0 ppm). ^{1}H and ^{13}C NMR assignments were confirmed by 2D COSY, HSQC, HMBC, and NOESY experiments. Mass spectrometry analysis was performed with Maldi-quadrupole time-of-flight (Q-Tof) mass spectrometer equipped with Z-spray electrospray ionization source (ESI). Silica gel (200 mesh) was used for column chromatography. Analytical thin-layer chromatography was performed using silica gel (precoated sheets, 0.2 mm thick, 20 cm × 20 cm) and visualized by UV irradiation or molybdenum staining (heating with a phosphomolybdic acid reagent). DCM, MeOH, THF and toluene were dried over flame-dried 3 or 4 Å sieves. Dimethylformamide (DMF), triethylamine (Et3N), and trifluoroacetic acid (TFA) were used dry from Sure/Seal bottles. Other reagents were purchased from an industrial supplier. All UV reactions were carried out in a Luzchem photoreactor, LZC-EDU (110 V/60 Hz), containing 10 UVA lamps centered at 350 nm. TGA was conducted on a TA Q500 analyser, using a Pt pan as sample holder. After equilibrating the sample at 30 °C for 5 min and then at 100 °C for additional 20 min, the measurement was performed in air using a heating rate of 10 °C/min. The sample weight was monitored until 900 °C. The FTIR spectra were recorded with a Bruker Vertex 70v FTIR spectrometer equipped with a Germanium ATR accessory on solid samples. Absorption spectra were recorded on an Agilent Cary 8454 UV–vis diode array spectrophotometer. Fluorescence spectra were taken on a Horiba Jobin Yvon Fluoromax-4 spectrofluorometer in 1.00 cm × 1.00 cm quartz glass cells. DLS Measurements were performed on the Malvern Nano-ZS instrument operating in backscattering (173°) mode and analysed with the software Zetasizer, with automatic selection of the optimal detector position and number of independent measurements. Z-potential measurements were recorded on the same apparatus using the disposable Z-potential cuvettes. Confocal imaging was performed with a laser scanning confocal microscope equipped with a resonant scanner (Nikon A1R).

Section S2. Chemical Synthesis

Synthesis of functionalized fluorescein 2

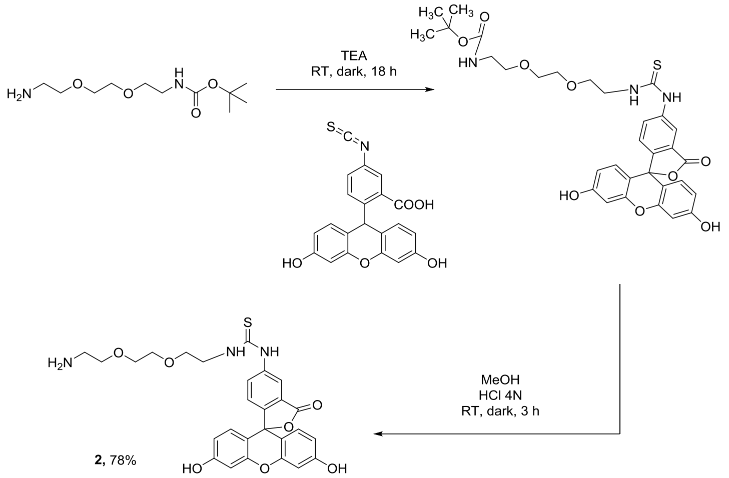

Scheme S1: Synthesis of functionalized fluorescein **2**.

Synthesis of functionalized fluorescein **2**. To a solution of Boc monoprotected 2,2′-(ethylenedioxy)bis(ethylamine) (166 mg, 0.67 mmol), in dry DCM (30 mL) was added FITC (200 mg, 0.51 mmol) in dry DMF (20 mL) and TEA (250 μL, 1.53 mmol) . The reaction mixture was stirred at room temperature, in the dark, for ca. 18 h. After reaction completion, the solvent was evaporated under reduced pressure and the crude product dissolved in MeOH (10 mL), and HCl 4 N in dioxane (5 mL) was added. The mixture was stirred at room temperature in the dark for 1 h, monitoring the reaction by TLC. The solvent was evaporated under reduced pressure and purification by flash chromatography (gradient elution in ethylacetate to methanol in ethylacetate) afforded the final product in 78% yield [1]. (orange solid). ^{1}H NMR (400 MHz, methanol-d4) δ 8.17 (s, 1H, Ar-H), 7.67 (d, *J* = 7.7 Hz, 1H, Ar-H), 7.01 (d, *J* = 8.2 Hz, 1H, Ar-H), 6.82 (d, *J* = 8.9 Hz, 2H, Ar-H), 6.70 (s, 2H, Ar-H), 6.58 (d, *J* = 8.8 Hz, 2H, Ar-H), 3.58 (s, *J* = 23.3 Hz, 2H, CH_2NHCS), 3.53 – 3.43 (m, 8H, CH_2O), 2.91 (s, 2H, CH_2NH_2). ^{13}C NMR (101 MHz, methanol-d4): δ 181.9 (C=S), 168.7 (C=O), 164.8, 155.7, 142.0 (C-Ar), 130.8, 129.2, 126.9, 115.6, 113.16, 102.4 (CH-Ar), 70.3, 69.1, 66.8 (CH_2OCH_2), 44.1 (CH_2)NHSNH, 39.5 (CH_2NH_2); HRMS (ES-) m/z calcd for $C_{27}H_{27}N_3O_7S$ $[M - H]^-$ 537.1570, found 536.1497.

Synthesis of glycopeptide 1

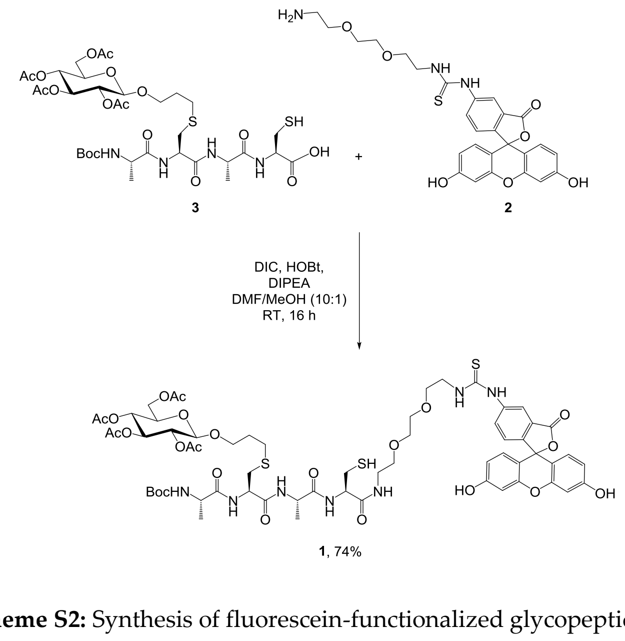

Scheme S2: Synthesis of fluorescein-functionalized glycopeptide **1**.

Synthesis of fluorescein-functionalized glycopeptide **1**. To a solution of **2** (50 mg, 0.06 mmol) in DMF (10 mL), was added HOBt (9.5 mg, 0.07 mmol) and DIC (7μL, 0.07 mmol) and the reaction was stirred at 0 °C for 1 h. The reaction was brought to room temperature and glycopeptide **3** (323 mg, 0.06 mmol) and DIPEA (160.3 mg, 1.24 mmol) in a mixture DMF:MeOH (10:1) was added and stirred for 16 h. The solvent was removed *in vacuo* and purification by flash chromatography (gradient elution in ethylacetate to methanol in dichloromethane) afforded the final compound as a yellow solid in 74% yield. IR 3305.44 (OH), 2925.25 (NH), 2853.96 (SH), 1747.76, 1658.09 (C=O), 1452.39 (SCH_2), 1368.44 (C=S), 1225.24 (CH Alkyl), 1058.05 (COC), 900.01, 850.68 (CH-Ar). ^1H NMR (400 MHz, MeOD) δ 8.13 (s, 1H, Ar-H), 7.76 (s, 1H, Ar-H), 7.13 (d, *J* = 8.2 Hz, 1H, Ar-H), 6.66 (dd, *J* = 13.8, 5.3 Hz, 4H, Ar-H), 6.52 (dd, *J* = 8.7, 2.3 Hz, 2H, ArCH), 5.35 (s, *J* = 3.0 Hz, 1H, H4), 5.17 – 4.98 (m, 4H, H-1,H-2,H-3,H-5), 4.60 (s, 2H, $\underline{CH_2}OCH_3$), 4.09 (m, *J* = 7.2 Hz, 4H, $\underline{CH}CH_3$, $\underline{CH}CH_3$, $\underline{CH_2}NO$), 3.84 (d, *J* = 27.4 Hz, 2H, $O\underline{CH_2}CH_2$), 3.75 – 3.60 (m, 8H, OCH_2), 3.60 – 3.48 (m, 2H, $\underline{CH_2}NS$), 3.37 (s, 1H, $CHCH_2SH$), 3.10 (s, 1H, $\underline{CH}CH_2S$), 2.90 (s, 2H, $CH\underline{CH_2}SH$), 2.86 – 2.72 (m, 2H, $CH\underline{CH_2}S$), 2.58 (m, 2H, $OCH_2\underline{CH_2}$), 2.11, 2.02, 1.99, 1.91 (s, 12H, $COCH_3$), 1.42 (s, 12H, $C(CH_3)_3$, $CH\underline{CH_3}$), 1.31 (s, 3H, $CH\underline{CH_3}$). ^{13}C NMR (101 MHz, MeOD/$\underline{CH_3COOH\text{-}d}$) δ 179.67 (C=S), 173.1, 172.2, 170,3, 169.3, 168.92, 168.82, 168.33, 168.18, 167.98, 167.44 (C=O), 158.16, 150.94, 146,51, 139.11, 128.50, 127.12, 125.66, 122.59, 116.22, 110.41 (Ar-C), 108.23 (C1), 100.33, 99.06 (Ar-C), 77.55 ($\underline{C}(CH_3)_3$, 69.12 (C3), 68.53 (C5), 68.29 (C2), 68.09, 67.35 (OCH_2), 67.27 ($O\underline{C}H_2$), 66.02 (C4), 65.62 ($\underline{C}HCH_2S$), 64.45 (C6), 61.58 ($\underline{C}HCH_2SH$), 59.36 ($\underline{C}HCH_3$), 55.13 ($\underline{C}HCH_3$), 42.19 ($\underline{C}H_2NS$), 39.90 ($\underline{C}H_2NO$), 37.23 ($S\underline{C}H_2CH$), 29.92 ($S\underline{C}H_2CH_2$), 27.41 ($OCH_2\underline{C}H_2$), 26.18 ($CH\underline{C}H_2SH$), 25.41 ($C(\underline{C}H_3)_3$), 22.03, 22.03, 22.03, 20.49 ($C(O)\underline{C}H_3$), 19.39, 17.77 ($CHCH_3$). $[\alpha]D^{26}$ = (deg cm^3 g^{-1} dm^{-1}) = 117° (c = 0.000145, MeOH). HRMS (MALDI) m/z calcd for $C_{61}H_{79}N_7O_{23}NaS_3$ $[M + Na]^+$ 1396.4287, found 1396.4326.

Synthesis of maleimido-PEG conjugate 4

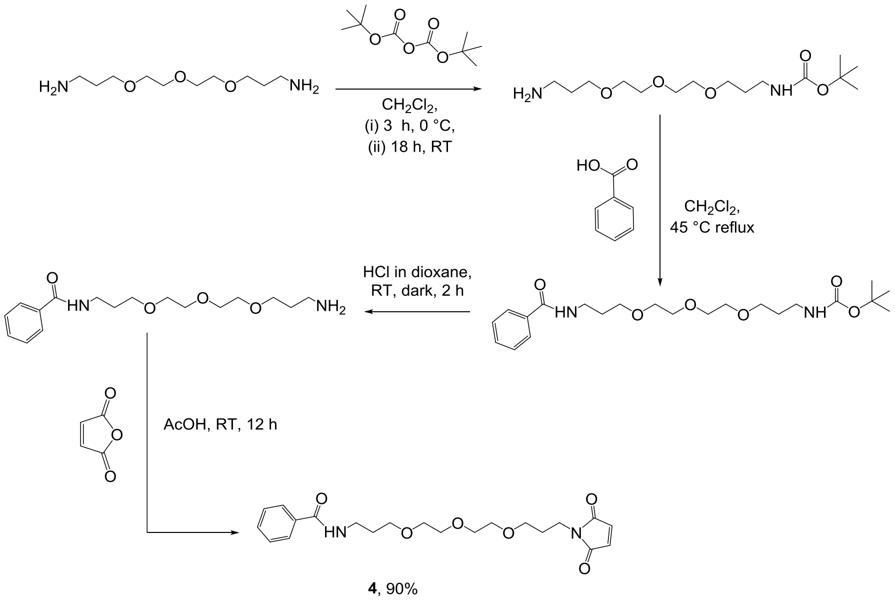

Scheme S3: Synthesis of Maleimido-PEG conjugate **4**.

Synthesis of maleimido-PEG conjugate **4**. To a solution of 4,7,10-trioxa-1,13-tridecanediamine (10 g, 0.045 mol) in CH_2Cl_2 (40 ml) in an ice bath, a solution of Boc_2O (5 g, 0.023 mol) in CH_2Cl_2 (40 mL) was added dropwise over a period of 3 h. The reaction mixture was stirred overnight at room temperature. The solvent was removed under reduced pressure and after mixing the residue with H_2O (40 ml) the precipitate formed was filtered on celite. The filtrated H_2O was extracted with CH2Cl2 (20 mL per 5), the organic phase was washed with H_2O (40 mL), brine (40 mL) and dried over Na_2SO_4. The evaporation of the solvent afforded the Boc-monoprotected compound as a

colorless oil (83%). Benzoic acid (1.90 g, 0.016 mol) were dispersed in 200 ml CH_2Cl_2 containing Boc-monoprotected PEG (5 g, 0.016 mol). After that, DMAP and EDC HCl (3.07 g, 0.016 mol) were added and the reaction mixture was stirred under reflux at 45 °C for 24 h. The solvent was evaporated under reduced pressure and once the residue was dispersed in CH_2Cl_2, it was washed with Na_2HCO_3 (20 mL per 4), HCl 1M (20 mL per 4), H_2O (40 mL), brine (40 mL) and dried over Na_2SO4. The evaporation of the solvent afforded the compound as a colorless oil (80%). The obtained compound was dissolved in 4M HCl in dioxane (5mL) and the mixture was stirred at room temperature in the dark for ca. 1 h monitoring by TLC. The solvent was evaporated under reduced pressure and the product was dried under vacuum overnight (100%). To that compound (4.66 g, 0.014 mol) a solution of maleic anhydride (1.41 g, 0.014 mol) in 15 mL of glacial AcOH were added under vigourous stirring. The solution was stirred for 12 h under nitrogen at R.T. The solvent was evaporated under reduced pressure and the product was dried under vacuum overnight. The crude was dispersed in 10 mL of H_2O and heated under reflux for 1 hour [2]. The evaporation of the solvent afforded the compound as a white oil (90%). ^1H NMR (400 MHz, MeOD) δ 7.83 – 7.78 (d, 2H), 7.56 – 7.48 (t, 1H), 7.45 (t, *J* = 7.4 Hz, 2H), 6.29 (s, 2H), 3.66 – 3.57 (m, 10H), 3.47 (t, *J* = 7.0 Hz, 2H), 3.30 (t, *J* = 3.2, 1.5 Hz, 2H), 3.14 – 3.02 (t, 2H), 1.96 – 1.83 (m, 4H). ^{13}C NMR (101 MHz, $CDCl_3$) δ 168.45, 167.78 (C=O), 133.82, 132.72, 131.67, 128.54, 127.37 (C-Ar), 70.15, 69.93, 69.74, 69.65, 69.41, 39.84 (OCH_2), 38.05, 29.09 (CH_2N), 26.33, 26.33 ($OCH_2\underline{C}H_2$). HRMS ($APCI^+$) m/z calcd for $C_{21}H_{29}N_2O_6$ $[M + H]^+$ 405.201818, found 405.202013.

Synthesis of Mal-CNOs

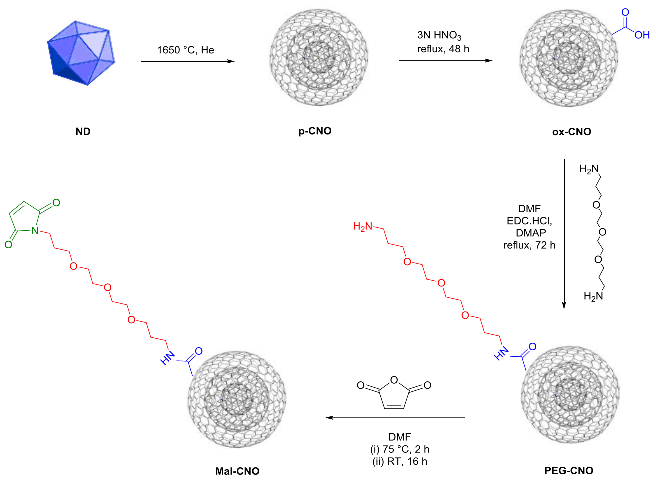

Scheme S4: Synthetic procedure for preparation of maleimido decorated CNOs.

Pristine CNOs (***p*-CNO**) were prepared by annealing nanodiamonds of 5 nm average particle size in a furnace at 1650 °C, according to the previously reported procedure.[1]

Subsequent oxidation of the *p*-CNO with nitric acid furnished **ox-CNO** with carboxylic acid groups on the surface, suitable for further ligation. Amidation with the diamino peg-linker, 4,7,10-trioxa-1,13-tridecanediamine using a water-soluble carbodimide coupling agent, 1-(3-thylaminopropyl)-3-ethylcarbodiimide hydrochloride and 4-(Dimethylamino)pyridine (DMAP) furnished **PEG-CNO**. Finally treatment with maleic anhydride furnished the maleimido decorated CNOs (**Mal-CNO**) suitable for covalent modification with cysteine containing biomolecules.

Section S3. UV Absorption/Emission studies and FTIR of glycopeptide

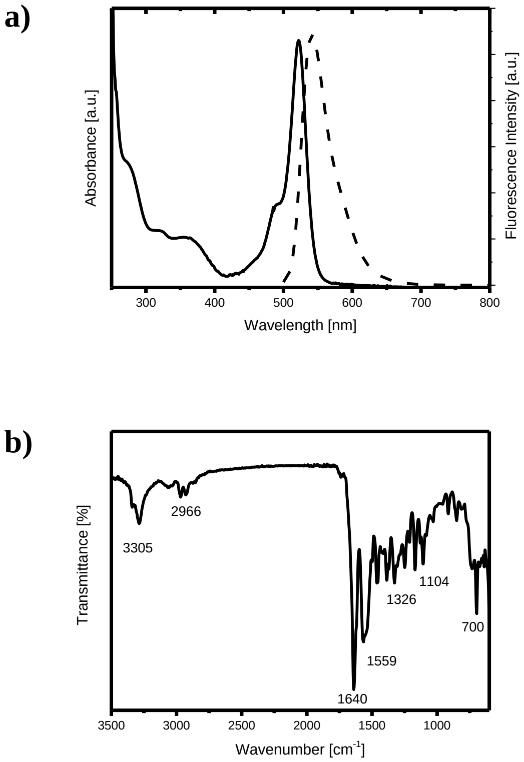

Figure S1: Absorption and Emission spectra (λ_{exc} = 495 nm) **(a)** of fluorescein-functionalized glycopeptide **1** at a concentration of 10 μg ml^{-1} in DMSO. FTIR spectroscopy **(b)** of synthetic glycopeptide **1**.

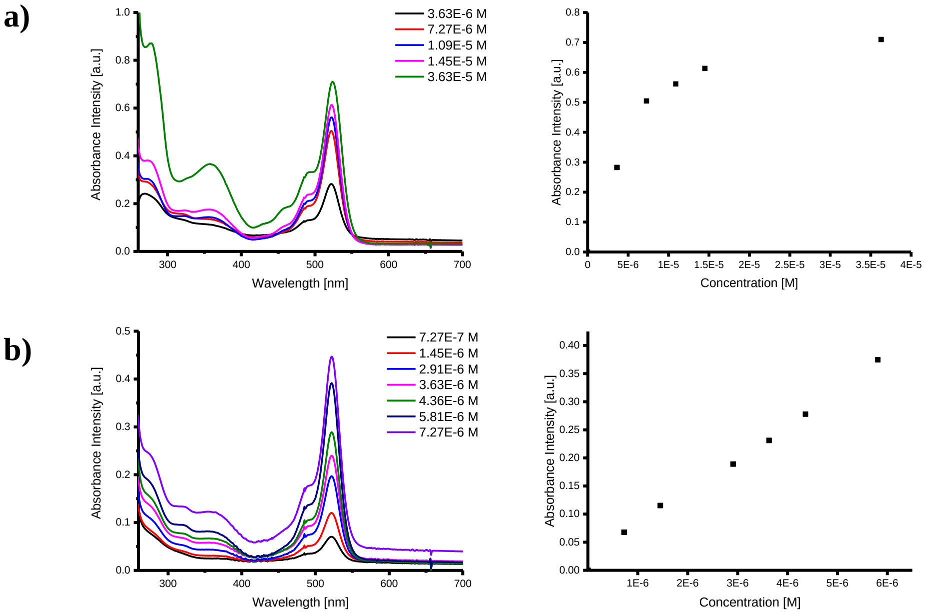

Figure S2: Absorption studies of fluorescent labelled glycopeptide **1** at a concentration of **(a)** 5, 10, 15, 20 and 50 μg mL^{-1} in DMSO and **(b)** 1, 2, 4, 5, 6, 8 and 10 μg mL^{-1} in DMSO (bottom).

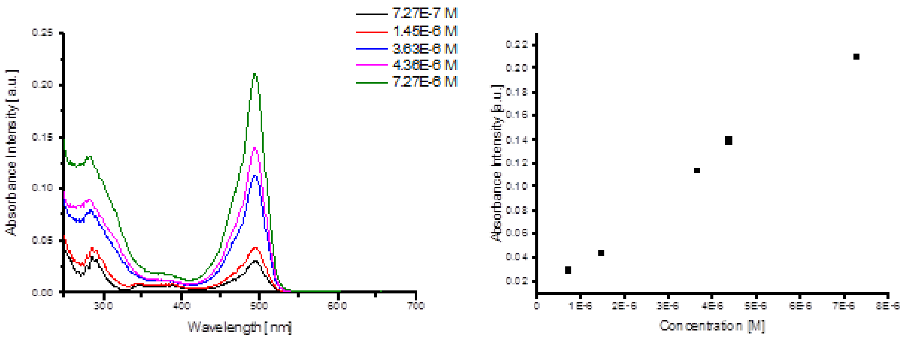

Figure S3: Absorption studies of fluorescent labelled glycopeptide **1** at a concentration of 1, 2, 5, 6 and 10 μg mL^{-1} in DMEM phenol red free.

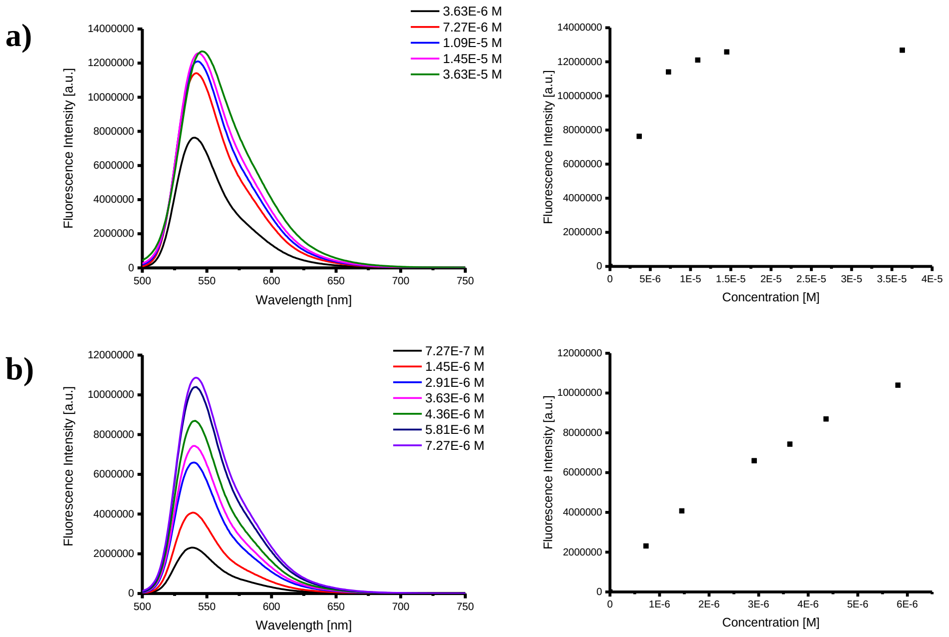

Figure S4: Emission studies of **(a)** fluorescent labelled glycopeptide **1** at a concentration of 5, 10, 15, 20 and 50 μg mL^{-1} in DMSO and **(b)** 1, 2, 4, 5, 6, 8 and 10 μg mL^{-1} in DMSO.

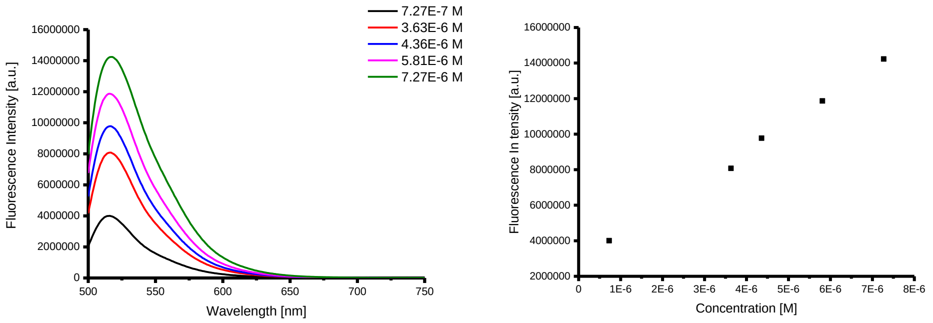

Figure S5: Emission studies of fluorescent labelled glycopeptide **1** at a concentration of 1, 5, 6, 8 and 10 μg mL^{-1} in DMEM phenol red free.

Section S4. Characterization of Functionalized CNOs

Absorption and emission studies of Gly-CNO

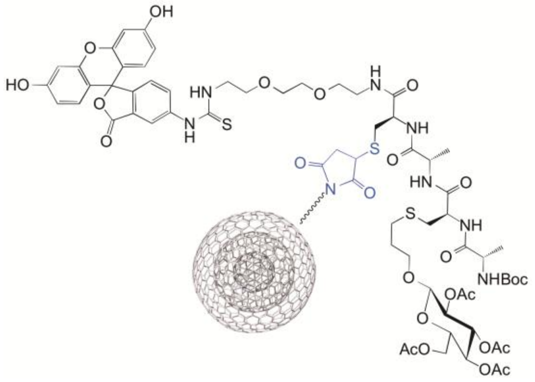

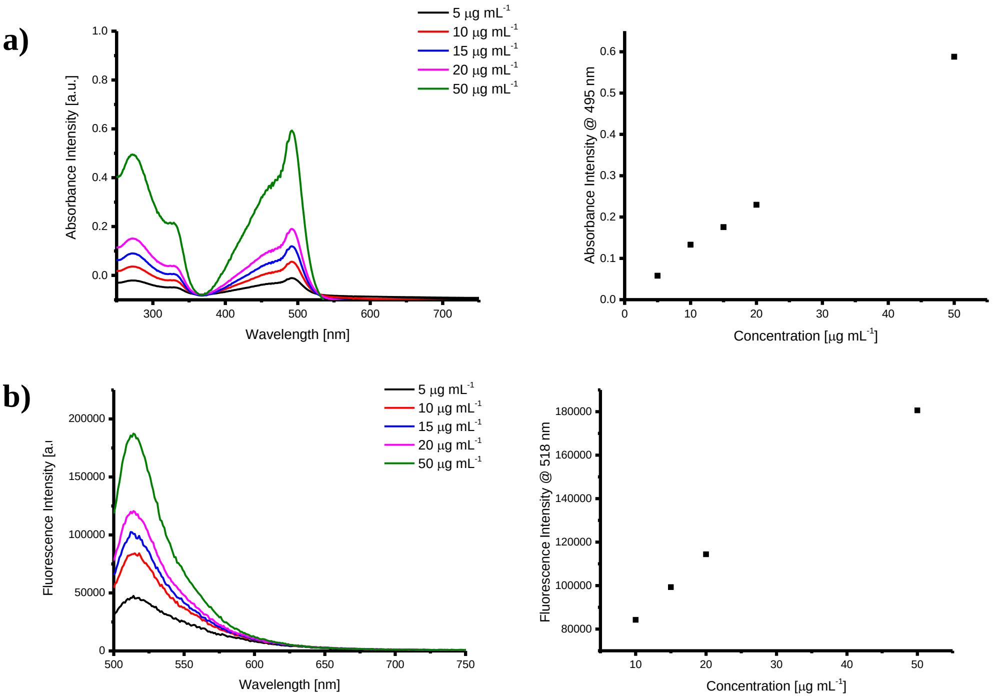

Figure S6: Absorption **(a)** and Emission spectra (λ_{exc} = 495 nm) **(b)** of **Gly-CNO** at 5, 10, 15, 20 and 50 μg mL^{-1} in DMEM-phenol red free.

Absorption and emission studies of BSA-CNO

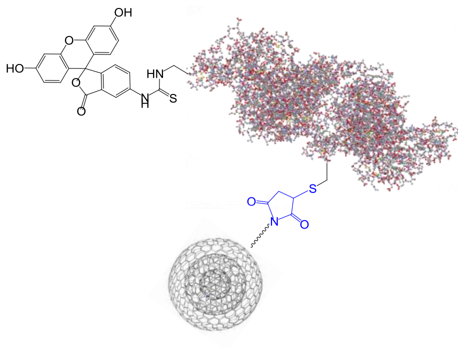

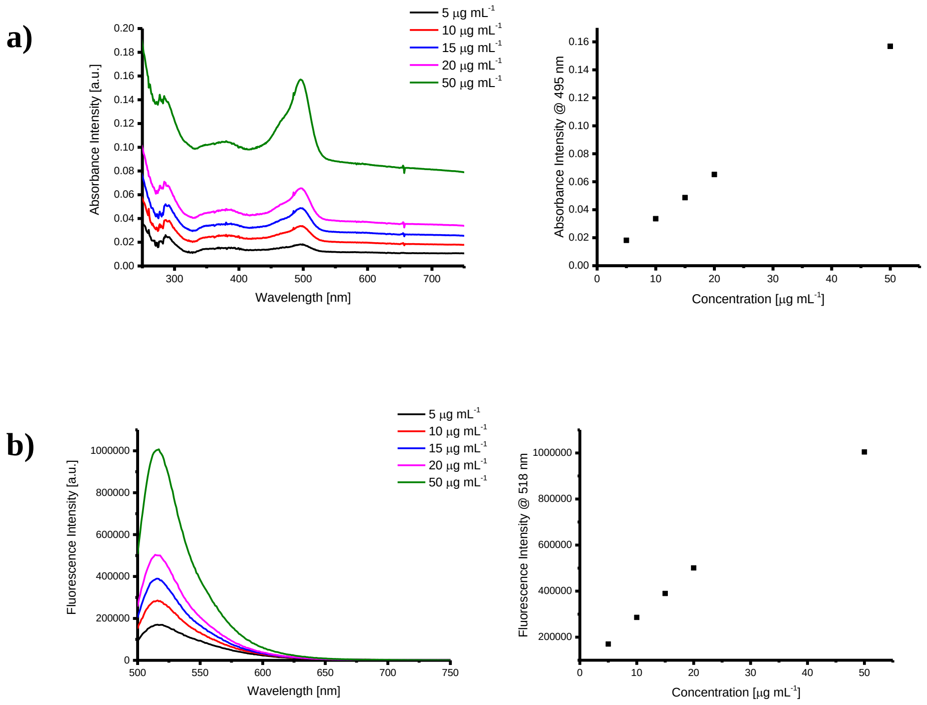

Figure S7: Absorption **(a)** and Emission spectra (λ_{exc} = 495 nm) **(b)** of **BSA-CNO** at 5, 10, 15, 20 and 50 μg mL^{-1} in DMEM-phenol red free.

DLS and Z-potential measurements of Gly-CNO and BSA-CNO

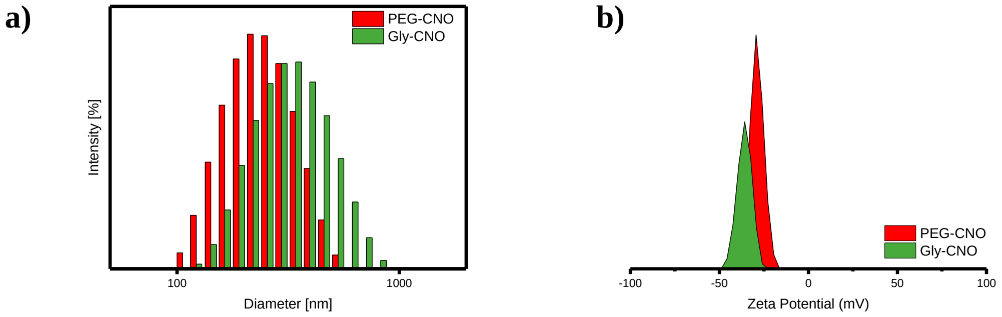

Figure S8: DLS **(a)** of **PEG-CNO** (red) and **Gly-CNO** (green) at a concentration of 5 μg ml^{-1} in water and Z-potential measurement **(b)** of **PEG-CNO** (red) and **Gly-CNO** (green) at a concentration of 10 μg ml^{-1} in phosphate buffer 0.01 M at pH 7.4.

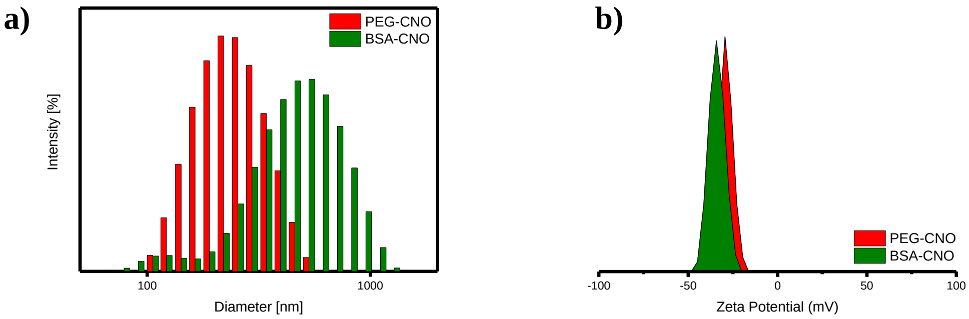

Figure S9: DLS **(a)** of **PEG-CNO** (red) and **BSA-CNO** (green) at a concentration of 5 μg ml^{-1} in water **(a)** and Z-potential measurement **(b)** of **PEG-CNO** (red) and **BSA-CNO** (green) at a concentration of 10 μg ml^{-1} in phosphate buffer 0.01 M at pH 7.4.

TEM of p-CNO, Gly-CNO and BSA-CNO

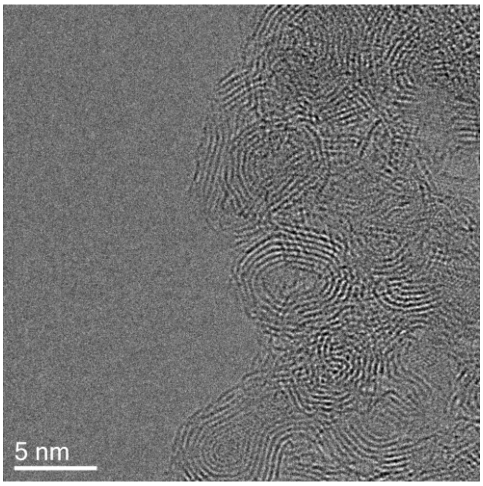

Figure S10. HR-TEM image of a portion of a cluster of pristine-CNOs, partly suspended on a hole in the carbon film.

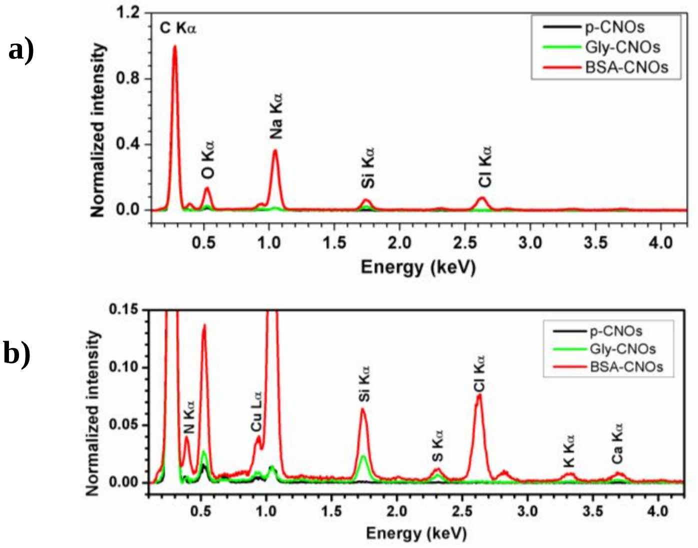

Figure S11. (a) STEM-EDS spectra acquired from portions of clusters of CNOs, suspended on holes in the carbon support film, from the samples p-CNOs, Gly-CNOs and BSA-CNO and **(b)** zoomed region of the same. The spectra are normalized to the C Kα peak. The Cu signal is due to the TEM grid, while the other signals, present in the Gly-CNOs and BSA-CNOs, are due to salts incorporated during sample treatment.

Section S5. NMR data

$^{1.}$ H and ^{13}C NMR of Fluorescein-Functionalized Glycopeptide (1)

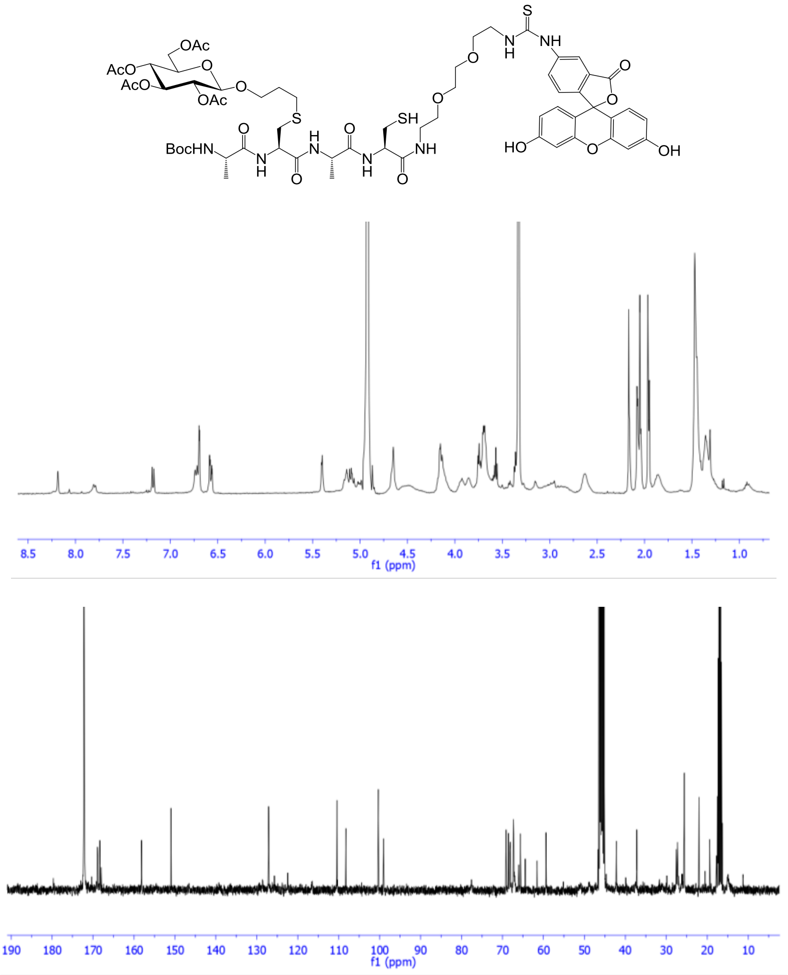

Figure S12. ^{1}H and ^{13}C NMR of Fluorescein-Functionalized Glycopeptide **1.**

^{1}H and ^{13}C NMR of Functionalized Fluorescein (2)

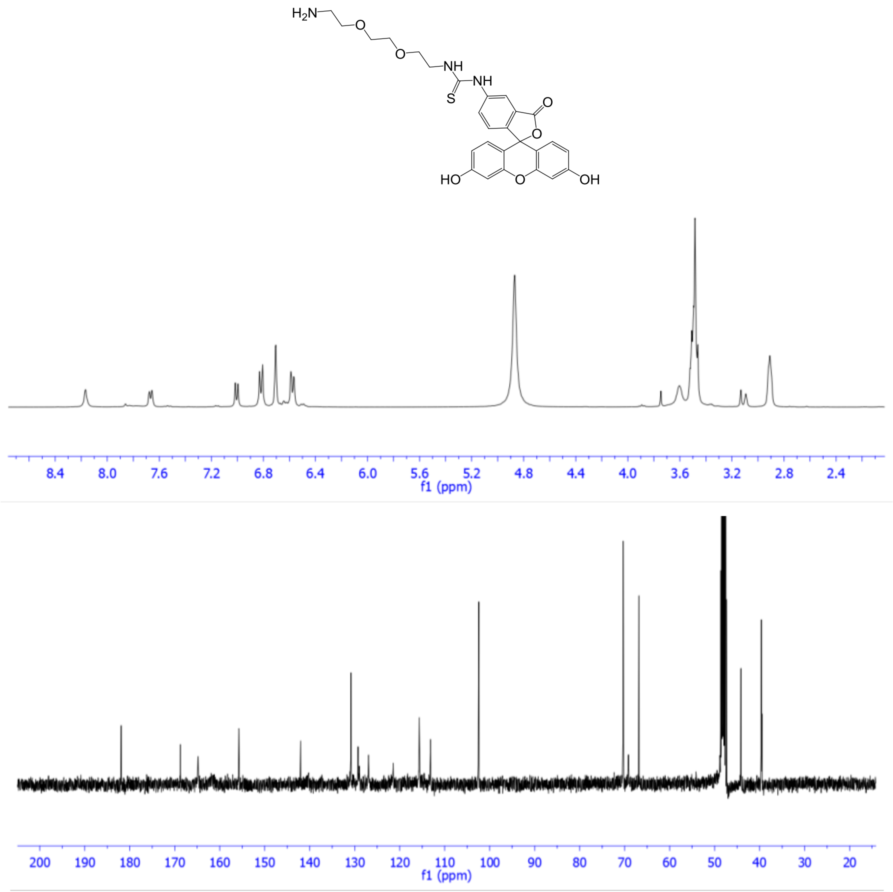

Figure S13. ^{1}H and ^{13}C NMR of Functionalized Fluorescein **2.**

^{1}H and ^{13}C NMR of compound 4

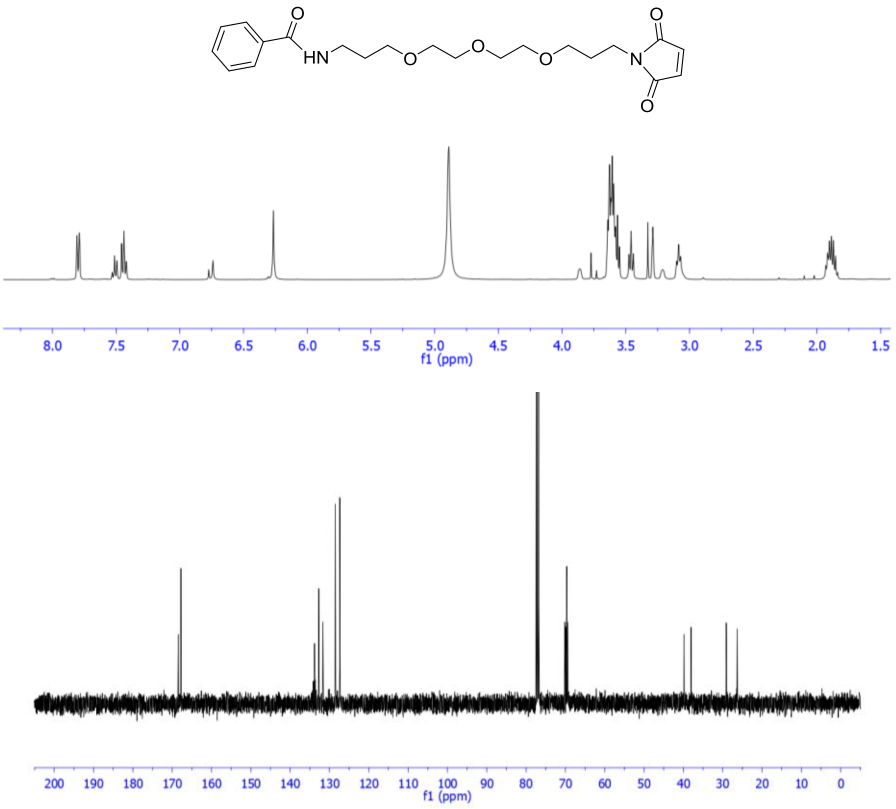

Figure S14. ^{1}H and ^{13}C NMR of compound **4.**

Section S6. HR-MAS of Mal-CNO

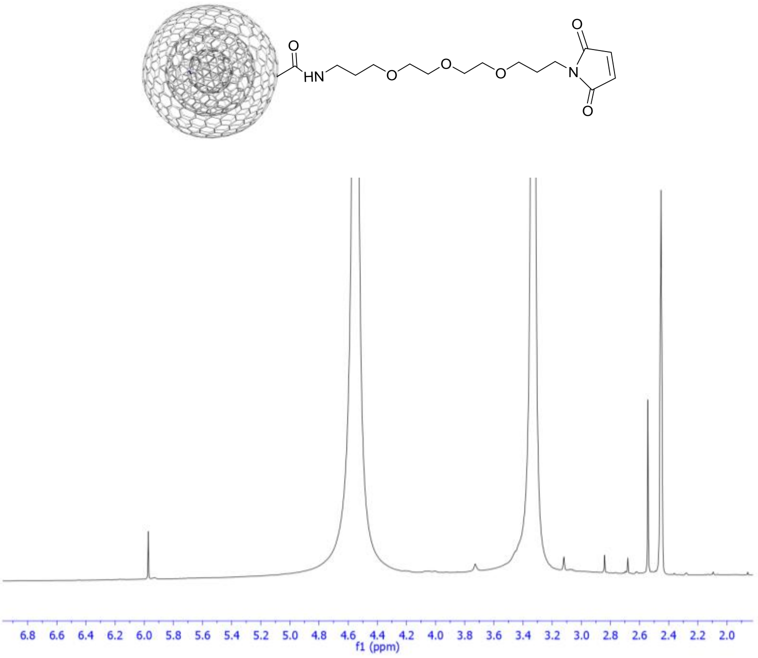

Figure S15. ^{1}H NMR of Mal-CNO.

Section S7. Cellular Uptake Experiments

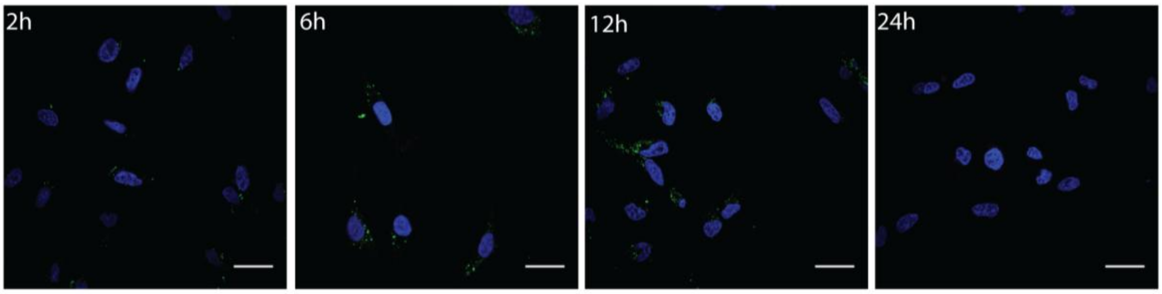

Figure S16. Fluorescent images of 3T3 cells incubated with **Gly-CNOs** at a mass concentration of 5 μg mL^{-1} for 2, 6, 12 and 24h. Nuclei are stained with Hoechst 33342 (blue). Scale bars = 20 μm.

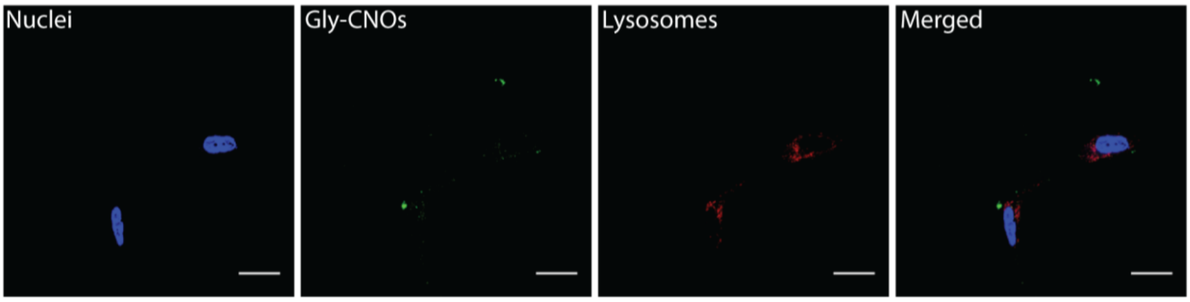

Figure S17. Fluorescent images of 3T3 cells incubated with **Gly-CNOs** at a mass concentration of 10 μg mL^{-1} for 2 h. Scale bars= 20 μm.

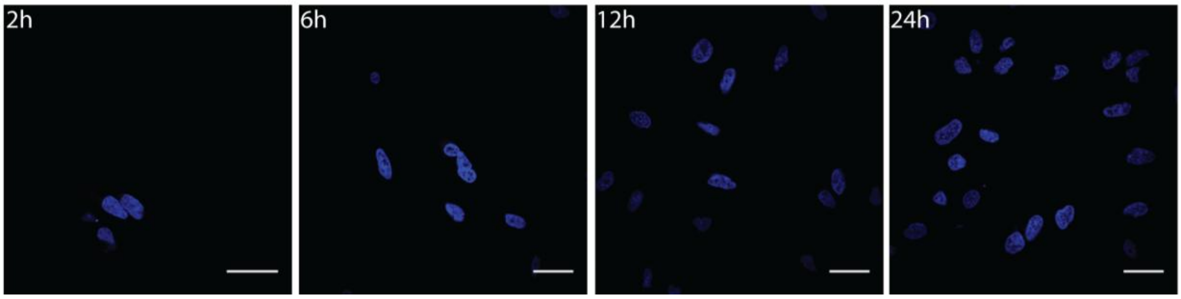

Figure S18. Fluorescent images of 3T3 cells incubated with glycopeptide **1** at a mass concentration of 20 μg mL^{-1} for 2, 6, 12 and 24h. Nuclei are stained with Hoechst 33342 (blue). Scale bars = 20 μm.

Section S8. Reference

1. Cioffi, C. T.; Palkar, A.; Melin, F.; Kumbhar, A.; Echegoyen, L.; Melle-Franco, M.; Zerbetto, F.; Rahman, G. M. A.; Ehli, C.; Sgobba, V.; Guldi, D. M.; Prato, M. *Chemistry - A European Journal* **2009**, *15* (17), 4419–4427.
2. Arosio, D.; Manzoni, L.; Araldi, E.M.V.; Scolastico, C. Cyclic rgd functionalized gold nanoparticles for tumor targeting. *Bioconjugate Chemistry* **2011**, *22*, 664-672.
3. Song, H.Y.; Ngai, M.H.; Song, Z.Y.; MacAry, P.A.; Hobley, J.; Lear, M.J. Practical synthesis of maleimides and coumarin-linked probes for protein and antibody labelling via reduction of native disulfides. *Organic & biomolecular chemistry* **2009**, *7*, 3400-3406.
